# Systemic Sclerosis Is Not Associated With Worse Outcomes of Patients Admitted for Ischemic Stroke: Analysis of the National Inpatient Sample

**DOI:** 10.7759/cureus.9155

**Published:** 2020-07-12

**Authors:** Ehizogie Edigin, Precious Eseaton, Subuhi Kaul, Hafeez Shaka, Pius E Ojemolon, Iriagbonse R Asemota, Emmanuel Akuna, Augustine Manadan

**Affiliations:** 1 Internal Medicine, John H. Stroger, Jr. Hospital of Cook County, Chicago, USA; 2 Internal Medicine, University of Benin, Benin City, NGA; 3 Anatomical Sciences, St. George's University, St. George's, GRD; 4 Rheumatology, John H. Stroger, Jr. Hospital of Cook County, Chicago, USA

**Keywords:** systemic sclerosis, scleroderma, ischemic stroke, cerebrovascular accident, cardiovascular, outcome, rheumatology

## Abstract

Introduction

Systemic sclerosis (SSc) is known to increase the risk of ischemic stroke and other cerebrovascular events. It is, however, unclear if SSc negatively impacts the outcomes of ischemic stroke hospitalizations. This study aims to compare the outcomes of patients primarily admitted for ischemic stroke with and without a secondary diagnosis of SSc.

Methods

Data were extracted from the National Inpatient Sample (NIS) 2016 and 2017 database. NIS is the largest hospitalization database in the United States. We searched the database for hospitalizations of adult patients admitted with a principal diagnosis of ischemic stroke, with and without SSc as the secondary diagnosis using International Classification of Diseases, Tenth Revision (ICD-10) codes. The primary outcome was inpatient mortality, and secondary outcomes were hospital length of stay (LOS), total hospital charge, odds of undergoing mechanical thrombectomy, and receiving tissue plasminogen activator (TPA). Multivariate logistic and linear regression analysis was used to adjust for confounders.

Results

Over 71 million discharges were included in the NIS database for the years 2016 and 2017. Out of 525,570 hospitalizations for ischemic stroke, 410 (0.08%) had SSc. Hospitalizations for ischemic stroke with SSc had similar inpatient mortality (6.10% vs 5.53%, adjusted OR 0.66, 95% CI (0.20-2.17); p=0.492), length of stay (LOS) (5.9 vs 5.7 days; p=0.583), and total hospital charge ($74,958 vs $70,197; p=0.700) compared to those without SSc. Odds of receiving TPA (9.76% vs 9.29%, AOR 1.08, 95% CI (0.51-2.27), P=0.848) and undergoing mechanical thrombectomy (7.32% vs 5.06%, AOR 0.75, 95% CI (0.28-1.98), P=0.556) was similar between both groups.

Conclusions

Hospitalizations for ischemic stroke with SSc had similar inpatient mortality, LOS, total hospital charge, odds of receiving TPA, and mechanical thrombectomy compared to those without SSc.

## Introduction

Systemic sclerosis (SSc), previously called scleroderma, is a complex rheumatologic disease characterized by immune-mediated vasculopathy, fibrosis of the skin, and internal organs, commonly the lungs and gastrointestinal tract [1-3]. The World Health Organization defined stroke as a clinical syndrome consisting of rapidly developing clinical signs of focal or global disturbance of cerebral function lasting more than 24 hours or leading to death with no apparent cause other than vascular origin [4]. Strokes are broadly classified as either ischemic strokes or hemorrhagic strokes. Ischemic strokes occur due to blood vessel obstruction with a resultant restriction in blood flow to the brain, whereas hemorrhagic strokes are due to a breach in blood vessels with extravasation of blood into the intracranial cavity [5]. Large national cohort studies have shown that SSc is independently associated with a higher risk of developing ischemic stroke [6-7]. Additionally, SSc is associated with increased incidence and prevalence of various cardiovascular (CV) diseases, such as pulmonary hypertension, acute myocardial infarction (MI), peripheral vascular disease, aortic and mitral regurgitation, in addition to cerebrovascular disease [8-9].

Given the fact that SSc causes a chronic inflammatory state, which may contribute to the increased risk of CV disease and stroke, it is unclear if the outcomes of ischemic stroke in patients with SSc differ from those without SSc. There is a scarcity of studies comparing ischemic stroke outcomes between patients with SSc and patients without SSc. To bridge this knowledge gap, we aimed at comparing outcomes of ischemic stroke hospitalizations with and without co-existing SSc using national-level population data. We also aimed to determine if ischemic stroke patients with SSc received different revascularization strategies compared to ischemic stroke patients without SSc. We used the two most recent releases of the Nationwide Inpatient Sample (NIS) database to address these clinically relevant issues.

## Materials and methods

Data source

We conducted a retrospective study of hospitalizations, in 2016 and 2017, with a principal diagnosis of ischemic stroke with and without a secondary diagnosis of SSc in acute-care hospitals across the United States (U.S). Hospitalizations were selected from the NIS database. Since this is aggregate level de-identified data, institutional review board approval was not sought.

The NIS was created and is maintained by the Agency for Healthcare Research and Quality and is the largest publicly available all-payer inpatient database in the U.S. It was designed as a stratified probability sample to be representative of all acute-care non-federal hospitals in the U.S. Hospitals are stratified according to ownership, geographic region, teaching status, urban/rural location, and bed size. A 20% probability sample of all hospitals within each stratum is then collected. All discharges from these hospitals are recorded and then weighted to ensure that they are nationally representative. The 2016 and 2017 NIS sampling frame includes data from 47 statewide data organizations (46 States plus the District of Columbia) that account for more than 97% of the U.S. population. Approximately, 30 discharge diagnoses for each hospitalization were recorded using the International Classification of Diseases, Tenth Revision (ICD-10) in NIS 2016, and 40 discharge diagnoses in the NIS 2017 database. In the NIS, diagnoses are divided into two separate categories: principal diagnosis and secondary diagnoses. The principal diagnosis is the main ICD-10 code for the hospitalization. Secondary diagnoses were any ICD-10 codes other than the principal diagnosis. As this data is representative of point prevalence of diagnoses, there is no method to distinguish between secondary diagnoses with an onset before the index hospital admission and those with an onset during the admission.

Inclusion criteria and study variables

The study population comprised all inpatient hospitalizations recorded in the NIS 2016 and 2017. Study variables included age, gender, race, hospital characteristics, medical comorbidities, and primary and secondary outcomes (outlined below). The following ICD-10 codes were used to identify principal and secondary diagnoses: for ischemic stroke, all I63 codes excluding I63.89 & I63.9, and for SSc all M34 codes (details in the supplementary table in the Appendix). Hemorrhagic stroke was excluded from the study. We studied baseline characteristics and outcomes for ischemic stroke hospitalizations in those with and without SSc.

Outcomes

The primary outcome was inpatient mortality. Secondary outcomes were hospital length of stay (LOS), mean total hospital charges, odds of receiving tissue plasminogen activator (TPA), and mechanical thrombectomy.

Statistical analysis

Analyses were performed using STATA, version 16 (StataCorp, Texas). A univariate logistic regression analysis using all variables and co-morbidities listed in Table 1 was used to calculate unadjusted odds ratios (ORs) for the primary outcome. All variables with P-values <0.1 were included in a multivariate logistic regression model. P-values <0.05 were considered significant in the multivariate analysis. Confounders were selected from the literature review. Charleston index was used to adjust for comorbidity burden. A multivariate logistic and linear regression model with all variables and co-morbidities listed in Table 1 were used to adjust for confounders for the secondary outcomes.

**Table 1:** Baseline characteristics of ischemic stroke hospitalizations with and without SSc SSc: Systemic sclerosis, MI: Myocardial infarction, PCI: percutaneous coronary intervention, CABG: Coronary artery bypass graft, COPD: Chronic obstructive pulmonary disease, DM: Diabetes mellitus, CHF: Chronic congestive heart failure, CKD: Chronic kidney disease, O2: Oxygen, Median household income: Median household income for patient’s Zipcode

	Ischemic stroke (n=525,570)		
	Without SSc (n=525,160)	With SSc(n=410)	P-value
Mean age (years)	70.3	65.5	0.004
Female	49.8%	84.2%	<0.0001
Race			0.050
White	70.3%	71.2%	Reference
Black	16%	13.7%	0.628
Hispanic	7.6%	5.5%	0.515
Asians	2.9%	5.5%	0.232
Charleston comorbidity index			0.0010
1	14.0%	0%	
2	13.0%	17.1%	
≥3	72.9%	82.9%	
Hospital bed size			0.4491
Small	13.6%	18.3%	
Medium	27.1%	25.6%	
Large	59.3%	56.1%	
Hospital teaching status			0.0547
Nonteaching	26.4%	17.1%	
Teaching	73.6%	82.9%	
Hospital location			0.0735
Rural	5.9%	1.2%	
Urban	94.1%	98.8%	
Expected primary payer			0.2770
Medicare	67.2%	74.1%	
Medicaid	9.2%	8.6%	
Private	19.8%	17.3%	
Self-pay	3.8%		
Median household income(quartile)			0.2779
1^st ^(0-25^th^)	30.0%	20.1%	
2^nd ^(26th-50^th^)	26.0%	33.3%	
3^rd^ (51st-75^th^)	24.2%	24.7%	
4^th^ (76th-100^th^)	19.8%	21.0%	
Hospital region			0.2985
Northeast	19.1%	24.4%	
Midwest	21.4%	25.6%	
South	41.0%	31.7%	
West	18.5%	18.3%	
Dyslipidemia	59.6%	43.9%	0.0036
Old MI	7.7%	4.9%	0.3438
Old PCI	0.73%	1.2%	0.6072
Old CABG	7.0%	0%	0.0131
Old pacemaker	3.5%	1.2%	0.2679
Atrial Fibrillation/flutter	30.8%	22.0%	0.0791
COPD	12.2%	9.8%	0.5076
Carotid artery disease	11.9%	11.0%	0.7997
Old stroke	8.6%	18.3%	0.0015
Hypertension	60.7%	51.2%	0.0755
Peripheral vessel disease	5.1%	2.4%	0.2696
Hypothyroidism	13.7%	26.8%	0.0006
DM type 1&2	36.5%	18.3%	0.0007
Obesity	12.4%	7.3%	0.1650
CHF	17.1%	19.5%	0.5699
CKD	17.3%	17.1%	0.9602
Liver disease	1.5%	4.9%	0.0129
Electrolyte derangement	20.3%	23.2%	0.5187
Maintenance hemodialysis	1.2%	2.4%	0.2895
O2 dependence	1.2%	4.8%	0.0024
Smoking	23.0%	20.7%	0.6244
Anemia	14.7%	17.1%	0.5354

## Results

There were over 71-million discharges included in the combined 2016 and 2017 NIS database. Out of 525,570 adult hospitalizations for ischemic stroke, 410 (0.08%) had SSc. The demographic characteristics of ischemic stroke hospitalizations with and without co-existing SSc are presented in Table 1.

We found that the patients in the SSc group were younger (65.5 vs 70.3 years, P=0.004) and composed of significantly more females (84.2% vs 49.8%, P<0.0001). The SSc group had less dyslipidemia (43.9% vs 59.6%, p=0.0036), diabetes mellitus (DM) (18.3% vs 36.5%, p=0.0007), and old coronary artery bypass graft (CABG) (0% vs 7.0%, p=0.0131) but had more old stroke (18.3% vs 8.6%, p=0.0015).

There were 29,025 (5.5%) ischemic stroke hospitalizations that resulted in in-hospital deaths. Of these, 25 deaths occurred in patients with SSc. We found that hospitalizations for ischemic stroke with SSc had similar inpatient mortality (6.1% vs 5.5%, AOR 0.66, 95% CI (0.20-2.17); p=0.492), LOS (5.9 vs 5.7 days; p=0.583), and total hospital charge ($74,958 vs $70,197; p=0.700) as compared to those without SSc. The odds of receiving TPA (9.76% vs 9.29%, AOR 1.08, 95% CI (0.51-2.27), P=0.848) and undergoing mechanical thrombectomy (7.32% vs 5.06%, AOR 0.75, 95% CI (0.28-1.98), P=0.556) was similar between both groups (Table 2).

**Table 2:** Clinical outcomes of ischemic stroke hospitalizations with and without SSc SSc: Systemic sclerosis, TPA: Tissue plasminogen activator, LOS: Hospital length of stay, C.I: Confidence interval

	Stroke with SSc (n=410)	Stroke without SSc (n=525,160)	Adjusted Odds Ratio (AOR)	P-value
	%	%	(95% CI)	
Primary outcome				
In-hospital mortality	6.1	5.5	0.66 (0.20-2.17)	0.492
Secondary outcomes				
TPA	9.8	9.3	1.08 (0.51-2.27)	0.848
Mechanical thrombectomy	7.3	5.1	0.75 (0.28-1.98)	0.556
			Adjusted mean difference	
LOS, mean, days	5.9	5.7	-0.31 ({-1.40}-0.79)	0.583
Total charge, mean $	74,958	70,197	-3,373 ({-20,527}-13,780)	0.700

## Discussion

There are different mechanisms by which SSc can potentially increase the risk of MI and stroke. Of the various mechanisms postulated, microvascular and macrovascular seem to be the most favored; however, uncertainty still exists as to which is the predominant underlying mechanism. The diverse mechanisms proposed include i) macrovascular abnormalities with consequently accelerated atherosclerosis, ii) microvascular pathology and endothelial dysfunction, with consequent oxidative stress, which can contribute to the observed accelerated atherosclerosis in patients with SSc, and iii) microvascular dysfunction independent of atherosclerosis [10].

It is well-known that chronic inflammation associated with rheumatologic diseases, such as rheumatoid arthritis (RA), systemic lupus erythematosus (SLE), and idiopathic inflammatory myopathy, is associated with premature atherosclerosis. Inflammatory cytokines, oxidative stress, and activated inflammatory cells have been shown to cause endothelial dysfunction and endovascular injury, which results in accelerated atherosclerosis and possibly increases the risk of ischemic stroke [11]. While a microvascular disease is a known characteristic of SSc, the presence and extent of macrovascular diseases and accelerated atherosclerosis in SSc patients remains a debated topic [9]. Previous studies have not shown conclusive evidence of the increased prevalence of traditional risk factors for CV disease in patients with SSc, and the distribution of these risk factors does not explain the increased risk of cerebrovascular diseases in patients with SSc [10,12-13]. We also know that inflammation can be a direct cause of arterial occlusion and may not be represented by traditional risk factor evaluation. Our study did not show an increase in traditional CV risk factors in the SSc group. Furthermore, the SSc cohort in our study had a lower prevalence of dyslipidemia, DM, and old CABG but had an increased history of old stroke. However, specifics of the old stroke (e.g., anti-phospholipid antibody syndrome status) are not available in the NIS.

Rheumatologic and dermatologic diseases with a prolonged systemic inflammatory state, such as SLE, ankylosing spondylitis, RA, and psoriasis, are known to increase the risk of stroke compared to the general population [14-15]. Studies have also demonstrated an increased risk of ischemic stroke in patients with SSc. A retrospective cohort study using U.S. veterans affairs administrative database records from 1999-2014, showed that the SSc cohort was at increased risk of developing ischemic stroke, with adjusted hazard ratio: HR 1.21 (95% CI 1.05-1.40) [7]. Hence, SSc was concluded to be an independent risk factor with stroke. Another national cohort study using data from the registry of catastrophic illness in Taiwan found that SSc patients have a 43% increase in the risk of ischemic stroke as compared to controls (95% CI 12%-83%, P =0.004) [6]. Also, SSc treatment did not change the risk of future ischemic stroke in SSc patients [6]. A meta-analysis of four retrospective cohort studies revealed that SSc patients have a statistically significant increase in ischemic stroke risk with a pooled risk ratio of 1.68 (95% CI, 1.26-2.24) [16]. However, despite the increased risk of developing a stroke, we found no difference in stroke outcomes, including inpatient mortality, LOS, and total hospital charge. Also, management was similar in both groups, in terms of the odds of undergoing TPA, and mechanical thrombectomy. Further national population-based studies are needed on this subject.

The strengths of this study are that it utilized information from the NIS, a large nationwide dataset with a large sample size that increases our study's power. In addition, the nature of the database allows us to compare the baseline demographic characteristics and various hospital outcomes between ischemic stroke hospitalizations with and without concomitant SSc.

There are several limitations to this study. Firstly, NIS database studies are subject to all the biases associated with retrospective studies. Second, the NIS is an administrative database that uses ICD-10 codes to characterize diagnoses and hospitalization events, hence, there is a possibility of errors associated with coding. Third, this report reflects data on ischemic stroke hospitalizations rather than on individual patients, therefore, individuals hospitalized multiple times with the same principal discharge diagnosis would be counted multiple times. Fourth, the reason for inpatient mortality is not available in the NIS. Lastly, data on immunosuppressant use and adherence, disease duration, treatment, the extent of disease, various specific organ involvement, type of SSc, and laboratory results, which could indicate underlying disease severity and inflammatory activity are not available in the NIS database.

## Conclusions

This retrospective analysis found that hospitalizations for ischemic stroke with SSc had similar inpatient mortality, LOS, total hospital cost, odds of receiving TPA, and mechanical thrombectomy as compared to those without SSc. Although SSc is known to increase the risk of ischemic stroke, hospitalizations for ischemic stroke with SSc had similar outcomes compared to those without co-existing SSc based on the U.S. NIS database.

## Appendices

**Table 3:** Supplementary table containing used ICD-10 codes SSc: Systemic sclerosis, MI: Myocardial infarction, PCI: Percutaneous coronary intervention, CABG: Coronary artery bypass graft; ICD-10: International Classification of Diseases, Tenth Revision

	ICD-10 codes
Diagnosis codes	
Ischemic stroke	All I63 codes excluding I63.89 & I63.9 codes
SSc	M34
Procedure codes	
Tissue plasminogen activator	3E03317
Mechanical thrombectomy	03CG3ZZ, 03CG0ZZ, 03CG3Z7, 03CG4ZZ
Comorbidities codes	
Dyslipidemia	E78
Old MI	I252
Old PCI	Z9861
Old CABG	Z951
Old pacemaker	Z950
Atrial fibrillation/flutter	I48
Chronic obstructive pulmonary disease	J41, J42, J43, J44
Carotid artery disease	I652
Old stroke	I63
Hypertension	I10
Peripheral vascular disease	I739
Hypothyroidism	E03
Diabetes mellitus type 1&2	E10, E11
Obesity	E660, E6601, E6609, E661, E662, E668, E669
Congestive heart failure	I50
Chronic kidney disease	N18
Liver disease	K70, K71, K72, K73, K74, K75, K76, K77
Electrolyte derangement	E870, E871, E872, E873, E874, E875, E876
Maintenance dialysis	Z992
Oxygen dependence	Z9981
Smoking	Z87891, F17200
Anemia	D50, D51, D52, D53, D55, D56, D57, D58, D59, D60, D61, D62, D63, D64
